# Epitaxially Grown Ultra-Flat Self-Assembling Monolayers with Dendrimers

**DOI:** 10.3390/molecules23020485

**Published:** 2018-02-23

**Authors:** Takane Imaoka, Noriko Bukeo, Kimihisa Yamamoto

**Affiliations:** 1 Laboratory for Chemistry and Life Science, Institute of Innovative Research, Tokyo Institute of Technology, Yokohama 225-8503, Japan; timaoka@res.titech.ac.jp (T.I.); norinya13@hotmail.com (N.B.); 2 ERATO, Japan Science and Technology Agency, Chiyoda 102-0076, Japan; 3 PRESTO, Japan Science and Technology Agency, Chiyoda 102-8666, Japan

**Keywords:** dendrimers, epitaxial growth, self-assembling monolayers, surface modification

## Abstract

Mono-molecular films formed by physical adsorption and dendrimer self-assembly were prepared on various substrate surfaces. It was demonstrated that a uniform dendrimer-based monolayer on the subnanometer scale can be easily constructed via simple dip coating. Furthermore, it was shown that an epitaxially grown monolayer film reflecting the crystal structure of the substrate (highly ordered pyrolytic graphite (HOPG)) can also be formed by aligning specific conditions.

## 1. Introduction

Self-assembled monolayers (SAMs) comprise a fundamental bottom-up nanotechnology technique widely used for applications such as interface control and solid surface functionalization. The most commonly used method for such bottom-up fabrication consists in SAM films utilizing thiol chemisorption on the Au substrate surface [1]. Despite being an extremely simple method that does not require any special equipment, it is robust and causes few defects (pinhole or line defect) under appropriate conditions [2,3]. However, there are limitations with these techniques in terms of the substrates and depositing materials. Therefore, generic SAM forming technology that can be used in all situations is in high demand. Here we present a new strategy of preparing such SAMs utilizing dendrimers.

## 2. Results and Discussion

**Figure 1:**
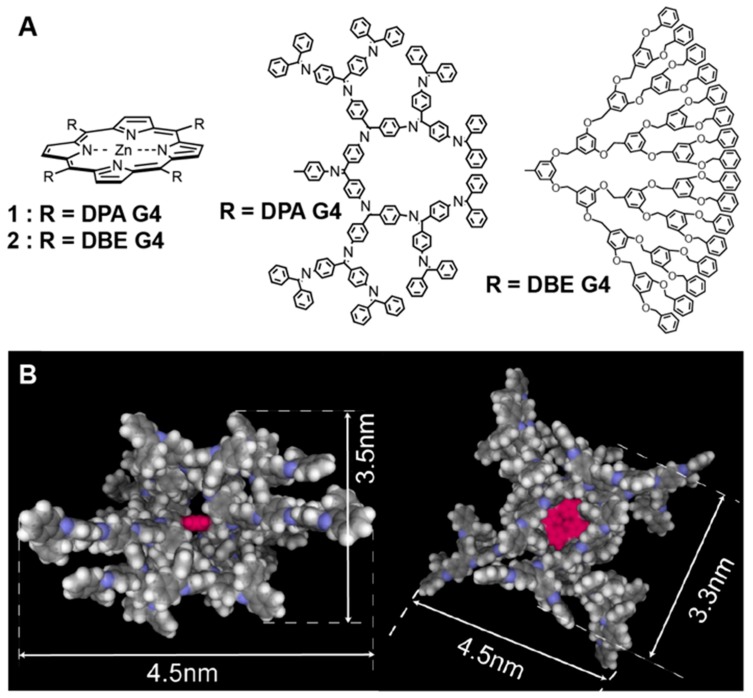
Structure of phenylazomethine dendrimer with a zinc porphyrin core (**1**). (**a**) Side view; (**b**) top view.

Although dendrimers are a class of macromolecules, they have a single molecular weight and a single structure [4,5,6]. Their compact structures allow for the formation of various higher order self-assembling structure owing to the weak and omnidirectional intermolecular interactions [7,8]. The material used in this study is phenylazomethine dendrimers with a zinc porphyrin core (**1**) [9,10,11]. Furthermore, this dendrimer has a capability for the precise accumulation for various metal ions [12] and organic molecules [13,14] in the interior. Therefore, it is a suitable molecule with many potential post-functionalization such as templated cluster synthesis [15,16,17,18,19], molecular shape recognition [11], anisotropic charge transfer [10,20,21,22], and further extension of the structural dimensions [23,24]. The dendrimer **1** (Figure 1) was synthesized according to a literature method [9] and deposited on the atomically flat surfaces of substrates including mica, HOPG (highly ordered pyrolytic graphite), and Au(111). We employed a dip-coating method to fabricate the dendrimer adlayer on these surfaces via immersion of **1** into a dilute solution (1 µmol L^−1^) in benzene for 1 min under ambient conditions.

**Figure 2:**
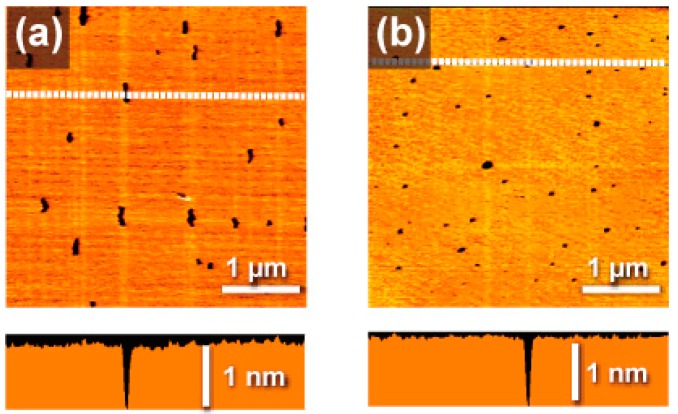
AFM (atomic-force microscopy) topographic images of the self-assembled monolayer (SAM) of **1** on mica. The fabrication was carried out through a dip-coating in a benzene (**a**) or toluene (**b**) solution of **1** (1 µmol L^−1^). Lower images are the cross section along the dashed lines shown in the topographic images.

Non-contact AFM (atomic-force microscopy) observations were carried out on the resulting surfaces modified with **1**, and topographic images are shown in Figure 2. Results of the mica substrates showed a highly coated flat surface modified with **1**, of which roughness was less than 0.1 nm, which is a level comparable to or higher than those previously reported [25,26]. Although a few pinholes were observed, the surface coverage factor was 98.8%, which can increase to 99.5% if toluene, instead of benzene, is used as the solvent. When the non-dehydrated grade of the solvent was used, the number and area of the pinholes increased. This means that pinholes are due to trace amounts of water in the solvents. The coverage of the SAMs was unexpectedly high. Even though SAMs via alkane thiols are well-established, many defects (holes) generally form on gold surfaces via immersion at room temperature [2,3]. In contrast, with this dendrimer, SAMs can be produced with low defects, even at room temperature. For the control experiment, a fourth-generation benzylether dendrimer (**2**) with the same zinc porphyrin core [27] and a similar molecular weight and hydrophobicity was employed. However, as opposed to monolayer films such as phenylazomethine dendrimers under the same conditions, many aggregates with a height of about 10 nm were observed (Figure S1).

These deposits are regarded as a monolayer of **1** because the maximum height of each step at the pinholes was ca. 1 nm. Because the step height of the surface layer observed at the place intentionally scratched in the contact mode was the same height of the pinhole, the pinhole was considered to have penetrated the substrate surface. The height of about 1 nm observed was shorter than the shorter axis of the dendrimer molecule and appeared to be inconsistent. However, as previously reported, it is not appropriate to approximate a dendrimer as a rigid sphere. It is natural to consider that the shrinking structure observed was a result of the repulsive force from the cantilever. When the concentration was reduced to 1/10, the depth remained at 1 nm, but the surface coverage drastically decreased (Figure S2). This means that a depth of 1 nm corresponds to one molecule of the dendrimer, which cannot be divided. On the other hand, when a dendrimer solution concentration one or two orders higher (10 or 100 µmol L^−1^) was used for the coating, the depth of the adlayer was increased to 2 or 4 nm, respectively (Figure S2). It is worth noting that the thickness of a smooth deposited layer at the molecular level can be freely changed at the level of 1 nm simply by changing the concentration of the solution. A similar SAM was also observed on highly oriented pyrolytic graphite (HOPG). XPS results indicating the presence of zinc atoms (Figure S3) on a modified surface of HOPG also proved the existence of **1**. In addition, cyclic voltammograms of the SAM-modified HOPG electrode (Figure S4) demonstrated that this assumption was valid. Total faradaic charge (0.62 µC) observed as one-electron oxidation of the zinc porphyrin core at 0.3 V vs. Ag/AgCl was equivalent to the calculated value (0.61 μC). The theoretical value was calculated on the assumption that a hexagonal close-packing monolayer of the dendrimer with a spacing of 4.4 nm formed on the electrode, the diameter of which was 9 mm. This spacing is based on a doubling of the hydrodynamic radii of the dendrimer.

**Figure 3:**
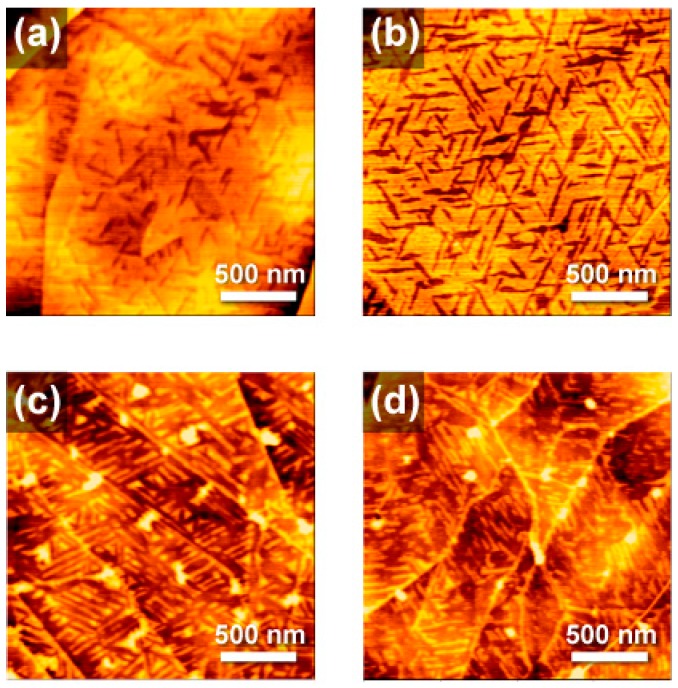
AFM topographic images of the SAM of **1** on highly oriented pyrolytic graphite (HOPG). The fabrication was carried out through dip-coating in a benzene solution of **1** (1 µmol L^−1^) in the absence (**a**) and presence of naphtalene (**b**–**d**). The concentration of naphtalene was 50 µmol L^−1^ (**b**), 100 µmol L^−1^ (**c**) and 200 µmol L^−1^ (**d**).

More interestingly, unique surface textures, as shown in Figure 3a, were occasionally found when the HOPG was used as the substrate for the benzene solution. The topography includes two different altitudes of terrace. The lower altitude sections that look like a valley were surrounded by a very flat 1 nm depth adlayer corresponding to the dendrimer monolayer. According to a higher magnification image (Figure S5), the lower layer is considered to be the surface of the HOPG because it is flat at the atomic level, whereas the higher layer is considered to be a layer of dendrimers since it shows slight irregularities.

Valleys in the monolayer lead to three specific directions. Angles between each direction were all 120° (or 60°), suggesting that the texture includes the regularity of *C*_3_ symmetry. Symmetric dendrimers including phenylazomethine dendrimers, like the carbon atoms in HOPG, are known to form a hexagonal close-packing (hcp) crystal on a flat surface. Based on this idea, we speculated that the surface monolayer formed through an epitaxial growth along the crystal structure of HOPG. However, it is not clear whether this mesophase structure was observed as an incompletion of the crystal growth or the thermodynamically stable structure.

To explain the origin of the geometric pattern, we examined different conditions of the fabrication. First, the immersion time was varied from 30 s to 10 min. However, no dependence on time was observed in the formation of the SAM of the dendrimer. Similarly, no variation was found when the concentration was reduced to 0.5 μmol L^−1^, indicating that the formation of the SAM reached an equilibrium condition. Therefore, we determined that the valleys originated from factors that prevented the adsorption of **1**.

Benzene molecules weakly adsorb to monocrystalline surfaces [28], including those of HOPG [29]. If solvent adsorption is a primary factor, this surface pattern should only appear with certain solvents such as benzene. For the control experiment, the same modification procedure was tested using toluene as the solvent. Because no characteristic pattern was found except for the flat dendrimer monolayer (Figure S6), the idea that the benzene molecule plays an important role in pattern formation was supported. This idea was further supported by additional experiments employing the other adsorbate. In particular, the naphthalene molecule was found to be a good candidate for the co-adsorbate. As shown in Figure 3b–d, the SAM prepared in the presence of naphtalene (100 µmol L^−1^) clearly showed the geometric pattern, and the texture was controllable by the concentration of naphtalene. When the concentration of naphtalene was increased to 200 µmol L^−1^, a significant decrease in the coverage ratio was shown, which was confirmed by lowering the bright area that the dendrimer SAM covered. In this case, discrete line-shaped islands (bright area) of the SAM 1 nm high were formed on the HOPG surface (dark area). A much lower concentration (50 µmol L^−1^) of naphtalene resulted in a higher coverage ratio, but the coverage was still lower than that formed without naphthalene. In this case, a periodic SAM that included many valleys formed. In any case, the islands or clacks were aligned in three specific directions, suggesting the epitaxial growth along the HOPG structure. In contrast to the case where naphthalene was used as a co-adsorbing molecule, no characteristic pattern was observed in any of the other extended π electron-based compounds. In these cases, only a simple monomolecular film was formed. This result suggests that the balance of adsorption energy of the dendrimer and the co-adsorption molecule to the substrate is important for the pattern formation.

## 3. Materials and Methods

The fourth-generation poly-phenylazomethine dendrimer (**1**) [9] and the poly-benzylether dendrimer (**2**) [27], both bearing a zinc porphyrin core, were synthesized according to a previously reported method. Other chemicals and solvents were purchased from Kantoh Kagaku Co. Ltd. (Tokyo, Japan). Natural mica substrates were purchased from Nilaco Co. (Tokyo, Japan), and the surfaces were cleaved just before use. Other substrates including HOPG (NT-MDT) and Au(111) on mica (Phasis) were purchased as commercial products.

The surfaces of mica and HOPG were cleaved just before processing. The dip-coating of dendrimers on a substrate was carried out by simply dipping the substrate into each dendrimer solution (solvent and concentration are shown in the main text) for 1 min. After the substrate was lifted out of the solution, excess solution was removed, and the sample substrates were dried under vacuum for 12 h at room temperature. The resulting surfaces were observed by non-contact atomic force microscopy (AFM) with a scanning probe microscope (SPI3800N, SII) using a standard silicon cantilever with Al coating (SI-DF-40P2, SII). Fast Fourier transform (FFT) processing was carried out using built-in software.

Electrochemical measurements were performed using a multipurpose electrochemical workstation (ALS-750b, CH Instruments, Austin, TX, USA). A vassal-plane carbon disk electrode (3.0 mm diameter purchased from BAS Inc., Tokyo, Japan.) was used as the working electrode. The electrode surface was polished with diamond and alumina paste and then rinsed in methanol with ultrasonication prior to use. Similar to the mica or HOPG substrates, the electrode surface was modified with the dendrimer **1**. An Ag/AgCl electrode in 3 mol L^−1^ NaCl_aq_ and a platinum coil were used as the quasi-reference and counter electrodes, respectively. Cyclic voltammetry (CV) and RDV measurements were conducted in an aqueous KPF_6_ solution (0.1 mol L^−1^), which was thoroughly bubbled with O_2_ or N_2_ gas prior to the measurements.

XPS were obtained using a spectrometer (JEOL, JPS-9000MC) with Mg Kα radiation. For the XPS measurement, gold powder was deposited on a glassy carbon substrate (Tokai Carbon Co., Ltd., Tokyo, Japan) as an internal standard with the cluster sample, and the Au 4*f*_7/2_ (84.0 eV) peak was used to offset the electron binding energy.

## 4. Conclusions

In summary, we have successfully prepared a dendrimer-based SAM on various substrates. The formation of a highly oriented texture pattern can be implemented through a simple dip-coating method. Employment of the co-adsorbate such as naphthalene, probably due to the epitaxy, can control this texture pattern. Although an example of epitaxial adsorption has been reported with respect to small organic molecules [30], this is the first epitaxy of a dendrimer to the best of our knowledge. The present results suggest that islands of the co-adsorbate molecule play a role as a guide for the patterning. Although the adsorption of benzene or naphtalene molecules to the HOPG surface is relatively weak, phase segregation between the packing structure of the dendrimer and the co-adsorbate should be considered an important factor for mesophase patterning. Similar to self-assembling phenomena reported elsewhere [31], a moderate balance between the intermolecular and interfacial adsorption energy might be essential for the production of a higher-order architecture.

## Supplementary Materials

Supplementary materials are available online.

## References

[1] Love J.C., Estroff L.A., Kriebel J.K., Nuzzo R.G., Whitesides G.M. (2005). Self-Assembled Monolayers of Thiolates on Metals as a Form of Nanotechnology. Chem. Rev..

[2] Poirier G.E. (1997). Characterization of Organosulfur Molecular Monolayers on Au(111) using Scanning Tunneling Microscopy. Chem. Rev..

[3] Yamada R., Sakai H., Uosaki K. (1999). Solvent Effect on the Structure of the Self-Assembled Monolayer of Alkanethiol. Chem. Lett..

[4] Tomalia D.A., Naylor A.M., Goddard W.A. (1990). Starburst Dendrimers: Molecular-Level Control of Size, Shape, Surface Chemistry, Topology, and Flexibility from Atoms to Macroscopic Matter. Angew. Chem. Int. Ed..

[5] Tomalia D.A., Khanna S.N. (2016). A Systematic Framework and Nanoperiodic Concept for Unifying Nanoscience: Hard/Soft Nanoelements, Superatoms, Meta-Atoms, New Emerging Properties, Periodic Property Patterns, and Predictive Mendeleev-like Nanoperiodic Tables. Chem. Rev..

[6] Tomalia D.A. (2010). Dendrons/dendrimers: Quantized, nano-element like building blocks for soft-soft and soft-hard nano-compound synthesis. Soft Matter.

[7] Rosen B.M., Wilson C.J., Wilson D.A., Peterca M., Imam M.R., Percec V. (2009). Dendron-Mediated Self-Assembly, Disassembly, and Self-Organization of Complex Systems. Chem. Rev..

[8] Percec V., Wilson D.A., Leowanawat P., Wilson C.J., Hughes A.D., Kaucher M.S., Hammer D.A., Levine D.H., Kim A.J., Bates F.S. (2010). Self-Assembly of Janus Dendrimers into Uniform Dendrimersomes and Other Complex Architectures. Science.

[9] Imaoka T., Tanaka R., Arimoto S., Sakai M., Fujii M., Yamamoto K. (2005). Probing Stepwise Complexation in Phenylazomethine Dendrimers by a Metallo-Porphyrin Core. J. Am. Chem. Soc..

[10] Imaoka T., Ueda H., Yamamoto K. (2012). Enhancing the photoelectric effect with a potential-programmed molecular rectifier. J. Am. Chem. Soc..

[11] Imaoka T., Kawana Y., Kurokawa T., Yamamoto K. (2013). Macromolecular semi-rigid nanocavities for cooperative recognition of specific large molecular shapes. Nat. Commun..

[12] Yamamoto K., Imaoka T. (2006). Dendrimer complexes based on fine-controlled metal assembling. Bull. Chem. Soc. Jpn..

[13] Ochi Y., Fujii A., Nakajima R., Yamamoto K. (2010). Stepwise Radial Complexation of Triphenylmethyliums on a Phenylazomethine Dendrimer for Organic–Metal Hybrid Assembly. Macromolecules.

[14] Ochi Y., Suzuki M., Imaoka T., Murata M., Nishihara H., Einaga Y., Yamamoto K. (2010). Controlled storage of ferrocene derivatives as redox-active molecules in dendrimers. J. Am. Chem. Soc..

[15] Yamamoto K., Imaoka T. (2014). Precision synthesis of subnanoparticles using dendrimers as a superatom synthesizer. Acc. Chem. Res..

[16] Yamamoto K., Imaoka T., Chun W.-J., Enoki O., Katoh H., Takenaga M., Sonoi A. (2009). Size-specific catalytic activity of platinum clusters enhances oxygen reduction reactions. Nat. Chem..

[17] Imaoka T., Kitazawa H., Chun W.-J., Yamamoto K. (2015). Finding the Most Catalytically Active Platinum Clusters with Low Atomicity. Angew. Chem. Int. Ed..

[18] Takahashi M., Koizumi H., Chun W.-J., Kori M., Imaoka T., Yamamoto K. (2017). Finely controlled multimetallic nanocluster catalysts for solvent-free aerobic oxidation of hydrocarbons. Sci. Adv..

[19] Imaoka T., Kitazawa H., Chun W.-J., Omura S., Albrecht K., Yamamoto K. (2013). Magic Number Pt_13_ and Misshapen Pt_12_ Clusters: Which One is the Better Catalyst?. J. Am. Chem. Soc..

[20] Imaoka T., Inoue N., Yamamoto K. (2012). Electron-transfer through potential gradient based on a dendrimer architecture. Chem. Commun..

[21] Imaoka T., Kobayashi H., Katsurayama M., Yamamoto K. (2015). A potential gradient along the layer-by-layer architecture for electron transfer rectification. Dalton Trans..

[22] Imaoka T., Inoue N., Yamamoto K. (2013). Extended Potential-Gradient Architecture of a Phenylazomethine Dendrimer. Org. Lett..

[23] Albrecht K., Hirabayashi Y., Otake M., Mendori S., Tobari Y., Azuma Y., Majima Y., Yamamoto K. (2016). Polymerization of a divalent/tetravalent metal-storing atom-mimicking dendrimer. Sci. Adv..

[24] Imaoka T., Bukeo N., Yamamoto K. (2015). A Self-Assembling Dendritic Reactor: Versatile Formation of Characteristic Patterns with Nanoscale Dimension. Macromol. Rapid Commun..

[25] Tsukruk V.V., Rinderspacher F., Bliznyuk V.N. (1997). Self-Assembled Multilayer Films from Dendrimers. Langmuir.

[26] Hierlemann A., Campbell J., Baker L., Crooks R.M., Ricco A.J. (1998). Structural Distortion of Dendrimers on Gold Surfaces: A Tapping-Mode AFM Investigation. J. Am. Chem. Soc..

[27] Pollak K.W., Leon J.W., Frechet J.M.J., Maskus M., Abruña H.D. (1998). Effects of Dendrimer Generation on Site Isolation of Core Moieties: Electrochemical and Fluorescence Quenching Studies with Metalloporphyrin Core Dendrimers. Chem. Mater..

[28] Wan L., Itaya K. (1997). In situ scanning tunnelling microscopy of benzene, naphthalene, and anthracene adsorbed on Cu(111) in solution. Langmuir.

[29] Chakarova-Kack S., Schroder E., Lundqvist B., Langreth D. (2006). Application of van der Waals density functional to an extended system: Adsorption of benzene and naphthalene on graphite. Phys. Rev. Lett..

[30] Miyamoto Y., Nemoto T., Yoshida K., Kurata H., Isoda S. (2004). In situ atomic force microscopy observation of the desorption process from monomolecular organic layers of a naphthalene derivative. Jpn. J. Appl. Phys..

[31] Elemans J.A.A.W., Lei S., de Feyter S. (2009). Molecular and Supramolecular Networks on Surfaces: From Two-Dimensional Crystal Engineering to Reactivity. Angew. Chem. Int. Ed..

