# Supplementary material for: Epitaxially Grown Ultra-Flat Self-Assembling Monolayers with Dendrimers
Source: Molecules. 2018 Feb 23;23(2):485. doi: 10.3390/molecules23020485 (PMC6017922; doi:10.3390/molecules23020485)
Supplement: Supplementary file 1 [file molecules-23-00485-s001.pdf]

Supplementary Information

Epitaxially Grown Ultra-flat Self-assembling Monolayer of Dendrimers

Takane Imaoka,[a,b,c] Noriko Bukeo[a] and Kimihisa Yamamoto*[a,b]

[a] Laboratory for Chemistry and Life Science, Institute of Innovative Research, Tokyo Institute of Technology, Yokohama 225-8503, Japan.
[b] ERATO and [c] PRESTO Japan Science and Technology Agency, Chiyoda 102-8666, Japan.

Supplementary Figures

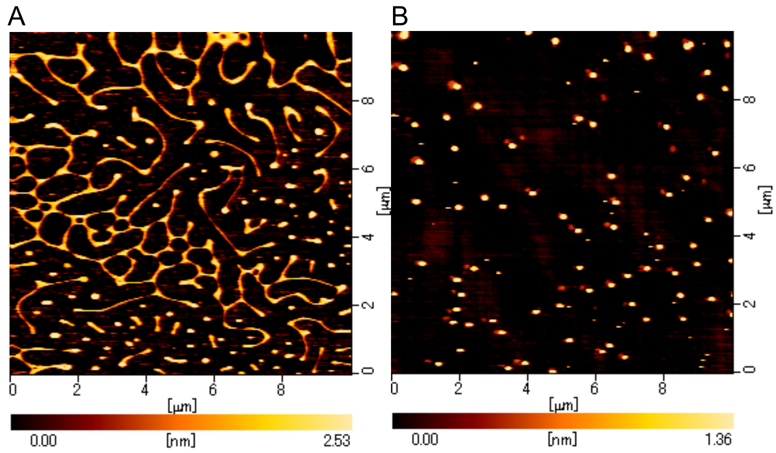

Figure. S1. AFM images of the A) Benzyl ether dendrimer (2), (B) Polyamidoamine dendrimer PAMAMG4OH purchased from Aldrich

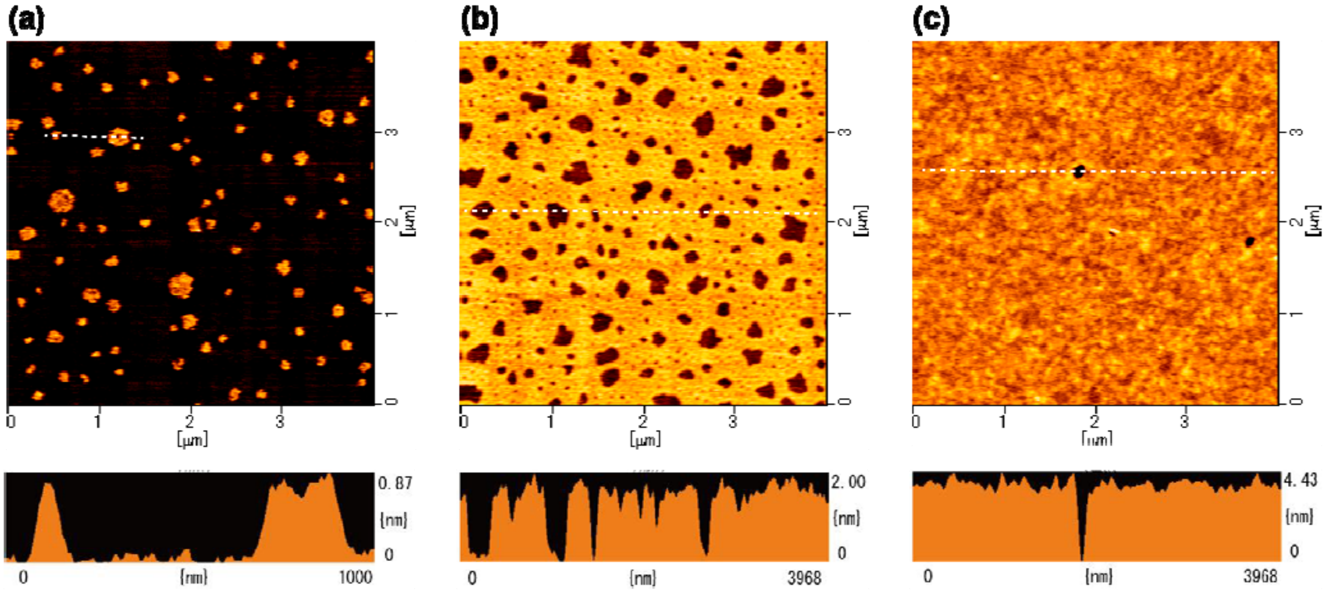

Figure. S2. AFM topographic images of the SAM of **1** on mica substrates. The fabrication was carried out by a dip-coating method using toluene as the solvent. The concentrations of **1** in the toluene solution were (a) 0.1 μmol L^{-1}, (b) 10 μmol L^{-1} and (c) 100 μmol L^{-1}, respectively. Lower images are the cross-section along the dashed lines shown in the topographic images.

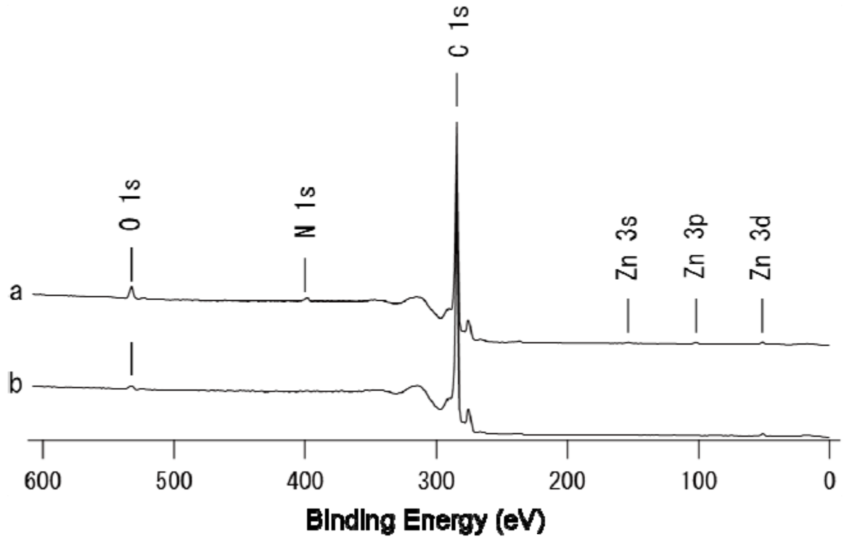

Figure. S3. XPS of HOPG substrates in the presence (a) and the abcence (b) of the SAM.

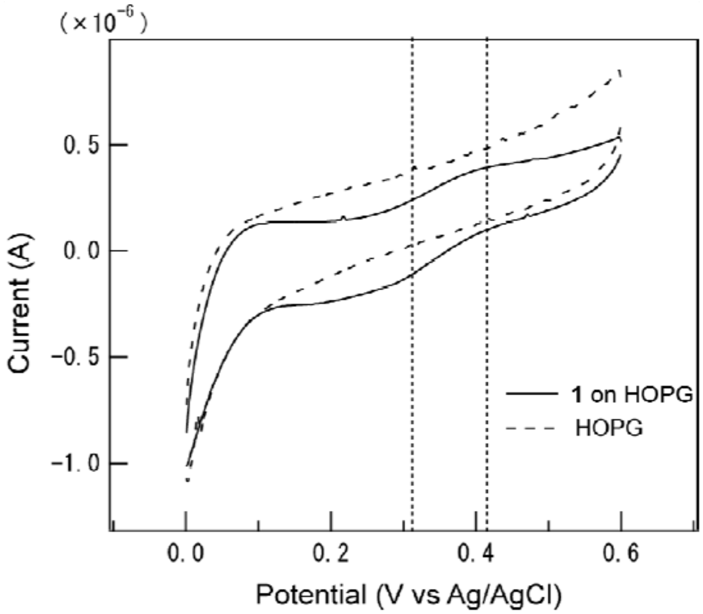

Figure. S4. Cyclic voltammograms of the dendrimer(**1**)-modified HOPG electrode and a bare HOPG electrode in aqueous solution of KPF_6 (0.1 mol L^{-1}).

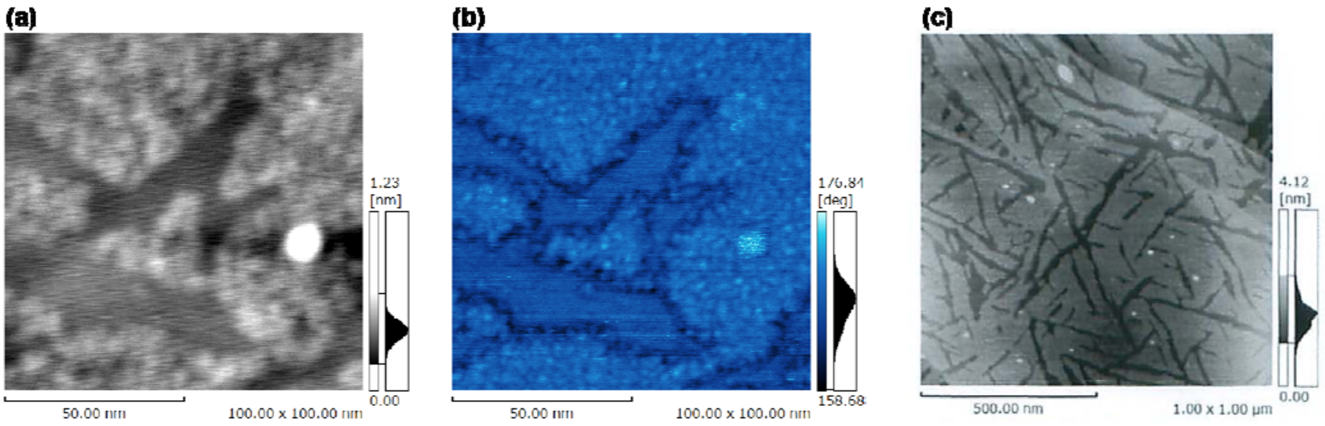

Figure. S5. (a) A higher magnification AFM topographic image and (b) a phase-shift image of the SAM of **1** on an HOPG substrate. (c) The lower magnification AFM topographic image at the same area. The SAM was prepared by dip-coating in a benzene solution of **1** (1 μmol L^{-1}) in the presence of naphthalene (50 μmol L^{-1}).

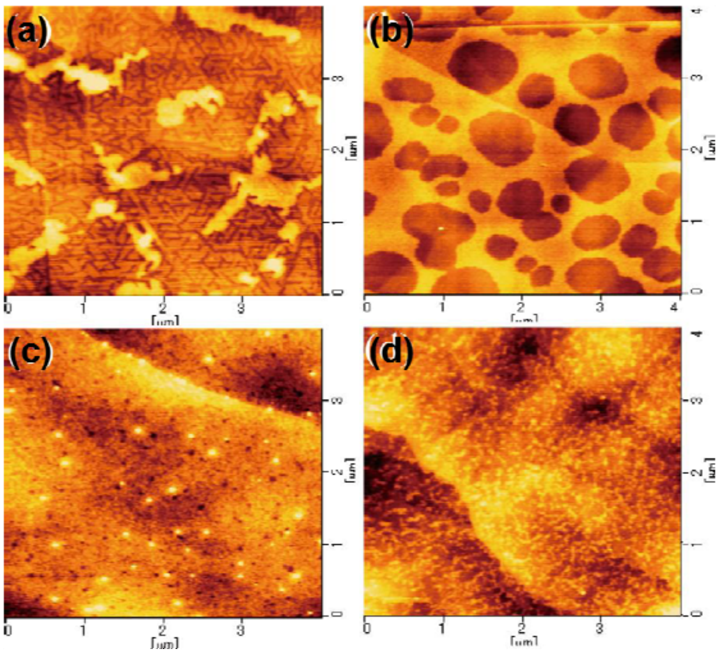

Figure. S6. AFM topographic images of the SAM of **1** on HOPG substrates. These SAMs were prepared by different solvents. (a) benzene, (b) chloroform, (c) tetrahydrofuran and (d) toluene.
